# COVID-19 in Assisted Living: Evidence on Policies, Provider Experiences, and Resident Mortality

**DOI:** 10.1093/geroni/igab046.216

**Published:** 2021-12-17

**Authors:** Kali Thomas, Lindsay Schwartz

## Abstract

The devastating effects of Coronavirus disease 2019 (COVID-19) among older adults residing in long-term care settings has been well documented. Assisted living settings in the U.S. have 811,000 residents; most are 80 years or older, and many have one or more chronic illnesses, making them highly susceptible to poor outcomes if exposed to COVID-19. This symposium highlights five studies that focus on various levels of COVID-19 response in assisted living: national organizations, states, assisted living operators and healthcare providers, and residents. The first study compares the sometimes conflicting guidance provided by national long-term care industry-related organizations and recommends assisted living-specific actions for the future. The second study describes the state regulatory response to COVID-19 in assisted living, identifying the themes and implications for the function of the care networks of assisted living residents. The third study presents findings from interviews with key stakeholders, including policymakers and industry leaders, that reflect on the challenges responding to changing recommendations and policies. The fourth study reports results from a survey with administrators and medical and mental health care providers who treat their residents that illustrates the care practices that were implemented in response to COVID-19 in assisted living. The fifth presentation documents the national excess assisted living resident mortality that was attributable to COVID-19. This symposium culminates with a leading assisted living industry expert reflecting on providers’ experiences and posing areas to consider when preparing for and responding to future pandemic events in assisted living settings.

